# Child, adolescent, and parent mental health in general population during a year of COVID-19 pandemic in belgium: a cross-sectional study

**DOI:** 10.1007/s44192-022-00019-w

**Published:** 2022-07-04

**Authors:** Amélyne Wauters, Julien Tiete, Joana Reis, Isabelle Lambotte, Simone Marchini, Véronique Delvenne

**Affiliations:** 1 Faculty of Medicine, Université Libre de Bruxelles, Brussels, Belgium; 2 Child and Adolescent Psychiatry Department, Queen Fabiola Children’s University Hospital, Brussels, Belgium; 3 Psychology Department, Erasme Hospital, Brussels, Belgium; 4 Faculty of Psychology and Education Sciences, Université Libre de Bruxelles, Brussels, Belgium; 5 Child and Adolescent Psychiatry Department, Erasme Hospital, Brussels, Belgium

**Keywords:** COVID-19, Lockdown, Child mental health, Depression, Anxiety

## Abstract

**Background:**

This study aims to evaluate the mental health status of children, adolescents and their parents during the first year of COVID-19 pandemic in Belgium.

**Method:**

Analysis compared results before and during the second national lockdown, which started on November 2nd 2020. A cross-sectional online survey was conducted between May 2020 and April 2021.

**Results:**

Two hundred and eighteen adults and 273 children fully completed the survey. Almost one in five children (17.9%) presented moderate-to-severe scores of depression. Adolescents presented a higher level of depression than children (p = 0.007). The rate of moderate-to-severe depression scores (10.8% to 21%, p = 0.007) and internalized symptoms increased during the second lockdown (p < 0.001). Parents' depression (p < 0.001) and anxiety (p = 0.027) levels also increased during the second lockdown. Logistic regression showed that the use of psychotropic medication in parents and parents’ depression scores were risk factors for children to have worse depression scores.

**Conclusion:**

The second lockdown appears to worsen the effects of the pandemic on children’s and parents’ mental health. There is a need to implement specific interventions targeting both children/adolescents and their parents to support them during lockdown periods and improve mental health outcomes.

## Background

More than two years after the discovery of the SARS-Cov-2 in late 2019, the COVID-19 pandemic is still the focus of discussions. Many questions are still pending about the management of beds in our intensive care units and medical specialist have been consulted to help making right decisions to relieve hospital congestion and support medical staff. In many countries, lockdown was the first solution to limit the spread of the virus. Schools and workplaces were closed; all non-essential activities and travel were forbidden. The epidemiologic impact of these national decisions brought quickly positive results but the psychological and developmental impact on children and adolescents were not initially considered. Many studies emerged to address these gaps. The largest and earliest study is certainly the one conducted in Hubei Province (China) between February and March 2020 [1]. Data showed an increase in depressive and anxious symptoms in children under 12 compared with baseline prevalence. In non-severely impacted areas during the first months of the outbreak, Yue et al. showed that children and their parent didn’t suffer major psychological distress [2]. Further survey regarding adolescents [3] showed worrying findings: 43.7% of the adolescents presented depressive symptoms and 37.4% anxious symptoms. Identified risk factors were being female and older. Liu et al. estimated that 34.8% of college students presented somatic symptoms related to concerns about the COVID-19 pandemic [4]. Researchers investigated suicide ideation and suicide attempts in the USA during the first wave of the pandemic. Data showed that 15.8% of adolescents reported suicide ideation and 4.3% did a suicide attempt during early 2020 [5]. Few studies have focused on the European population, using validated and suitable scales for children and adolescents. Most studies highlighted the deterioration of mental health in different countries in the world [6, 7]. Being a female, the lack of physical exercise and staying alone at home while parents are working were identified as risk factors [8, 9]. Problem-based coping strategy [9] and pandemic-related communication between children and parents [10] were also identified as protective factors for depression, anxiety and stress.

Social and health policy in Belgium to face the impacts of the pandemic were similar to those in other countries. On March 13th 2020, the Belgian government decided to close workplaces and schools, marking the beginning of the first lockdown. Throughout the year, federal decisions alternated between access to more individual freedom and tightening of restriction measures. On November 2nd 2020, the Belgian government decided on a second lockdown which lasted until the end of February 2021: contact professions had to stop working, cinema and enclosed areas had to close but schools remained open. However, the autumn holiday was extended to two weeks, such as the Easter vacation. The priority was to limit the spread of the virus and children were quickly singled out as a vector of the pandemic, particularly through school contacts. Canteens were closed and children had to remain with their schoolmates during playgrounds. Wearing a face mask became mandatory from the age of 10. High school students could only attend school one week out of the two since the beginning of the school year. They were allowed to go back full-time at school less than two months before the end of the school year, in May 2021.

To address mental health issues, teletherapy started to emerge in Belgium but also in other countries: although being an alternative solution to provide psychological care during quarantines, it didn’t seem appropriate for all clinical situations, especially among youth who presented deterioration in psychosocial functioning during lockdown [11]. Hospitalization was sometimes still needed but beds shortages remained a common issue during the pandemic, leading to “partial hospitalization at home” as a new attempt of finding solutions compatible with the deteriorating mental health of youth and the need to keep everyone at home. For some children, these alternatives were not enough, especially in cases of child abuse and eating disorders [12, 13].

Despite current recommendations on child and adolescent mental health [14], pediatric health professionals did not find that the needs of children and adolescents were truly taken into account in policy decisions. This might be explained by the lack of data. Indeed, working groups warned of the pressure and saturation of the mental health care system [15] but to our knowledge, no study has investigated mental health issues in Belgian children. This study aims to evaluate the mental health status of children and adolescents, aged from 7 to 17, during the first year of COVID-19 pandemic in Belgium and specifically before and after the second lockdown. November 2nd 2020 is a key date that corresponds to the federal decision to tighten health measures in Belgium and the start a second general lockdown. Parental levels of depression, anxiety and stress will also be assessed, as some authors found that youth mental health is closely tied to parental mental health during community-level stressors [16]. Because public policies were based almost exclusively on hospitalisation rates while the consequences on mental health (especially children’s) were not initially studied, our main hypotheses were that the prevalence of depression and anxiety in parents increased and could have consequences on children’s mental health.

## Methods

### Procedure

In this cross-sectional study, we conducted an online survey, completed simultaneously by a parent and their children. Inclusion criteria include the ability to speak French, parental age ≥ 18 years old and having children aged from 7 to 17 years. The questionnaire was distributed by official communication channels (hospitals websites, family associations, public health school) and others, via the snowball effect. The survey was set up on the secured REDCap^©^ (Research Electronic Data Capture) platform. Data on study participants have been stored on the REDCap^©^ database located on the leading site, Queen Fabiola Children’s University Hospital (HUDERF), Brussels, Belgium. The date of survey completion for each participant was automatically gathered by the REDCap^©^ system. Regarding participant’s safety, contact details of psychologists in our department were provided in case of need. Informed consent was obtained from all participants included in the study.

### Measures

The online survey was composed of two sections. The first section targeted parents. Sociodemographic information about themselves and their children, such as age, ethnic background, civil status, social support, financial impact of the pandemic, home location (urban/rural), parental educational level, psychiatric history and psychotropic medication were investigated.

Depression, Anxiety and Stress Scale (DASS-21) was used to assess parental depression and anxiety. DASS-21 is the short form of DASS-42, developed by Lovibond and Lovibond in 1995 [17], and has been studied in many countries and languages, including French [18]. Previous investigations showed significant correlations between DASS scores and the Beck Anxiety and Beck Depression scales [19]. This scale has proved an excellent internal consistency [20]. The 21-item version is divided into 3 sections of 7 items each: depression, anxiety and stress. Cut-offs can be used for depression (normal, 0–9; mild, 10–13; moderate, 14–20; severe, 21–27; and extremely severe, ≥ 28), anxiety (normal, 0–7; mild, 8–9; moderate, 10–14; severe, 15–19; and extremely severe, ≥ 20) and stress severity (normal, 0–14; mild, 15–18; moderate, 19–25; severe, 26–33; and extremely severe, ≥ 34).

The Child Behavior Checklist (CBCL) is a parental assessment of perceived child behaviors for children from 6 to 18 years old. Parents are asked to answer 113 questions with a 3 points Likert-scale: not true, sometimes true or often true. The French version was validated in the early 90’s and was studied on clinical and general population [21]. The psychopathology scores allow differentiating between a general population and a psychiatric population. Validation studies showed good test–retest and inter-judges liabilities [22]. We used the official computer software ADM (Assessment Data Manager program), using reference norms for Belgium, to calculate the results and distinguish internalized from externalized symptoms. Completing this questionnaire was optional, due to its high number of questions.

The second section targeted children and consisted of the French version of the 79-item Multiscore Depression Inventory for Children (MDI-C), a self-report scale adapted for children from 8 to 17 years old [23]. The MDI-C has strong internal consistency at the total score levels (0.92 to 0.94); therefore we used the total score instead of the subscales scores to evaluate children’s depression. The authors report good test–retest reliability.

### Participants

**Fig. 1:**
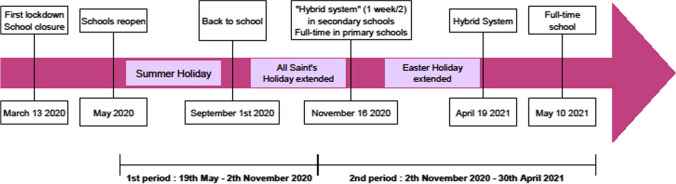
Chronology of restrictive measures taken to fight the COVID-19 pandemic, from an academic perspective

The survey was available from May 19, 2020 to April 30, 2021 (Fig. 1). Two thousand three hundred and thirty-nine people opened the online survey but only 199 families fully completed the survey by filling DASS-21, MDI-C and CBCL scales. Two hundred and nineteen families filled the DASS-21 and the MDI-C, allowing at least a double perspective from parents’ and children’s point of view. This low response rate (9.4%) might be explained by the length of the questionnaires approaching 45 min to fill them in completely. Many families included more than one child, with a total of 273 children completing the self-report scale (Table 1).

**Table 1:** Socio-demographic characteristics of participants

	Total		Before 2nd lockdown		During 2nd lockdown		*p*
	(n = 218 adults, 273 children)		(n = 69 adults, 85 children)		(n = 149 adults, 188 children)		
	N	%	N	%	N	%	
Demographic characteristics							
Children							
Age							
7–10 years old	82	29.7	34	40	48	25.5	0.057
11–13 years old	72	26.4	24	28.2	48	25.5	
14–17 years old	119	43.6	27	31.8	92	48.9	
Gender							
Boys	135	49.4	43	50.6	92	48.9	0.344
Girls	138	50.6	42	49.4	96	51.1	
Parents							
Age							
0–39 years old	46	21.1	16	23.2	30	20.1	0.267
40–45 years old	78	35.8	27	39.1	51	34.2	
46–51 years old	69	31.6	18	26.1	51	34.2	
51 +	25	11.5	8	11.6	17	11.4	
Ethnic origin							
Western europe	198	90.8	61	88.4	137	91.9	0.224
Eastern Europe	4	1.8	1	1.4	3	2	
South America	5	2.3	4	5.8	1	0.7	
Maghreb	3	1.4	2	2.9	1	0.7	
Other	8	3.7	1	1.4	7	4.7	
Financial impact							
None	121	55.7	41	59.4	80	53.7	0.058
Limited	62	28.3	21	30.4	41	27.5	
Significant	21	9.6	4	5.8	17	11.4	
Important	7	3.2	0	0	7	4.7	
Other	7	3.2	3	4.3	4	2.7	
Living environment							
Town	124	56.6	42	60.9	82	55	0.712
Countryside	94	43.3	27	39.1	67	45	
Civil situation							
Single	38	17.4	13	18.8	25	16.8	0.902
In a relationship	180	82.6	56	81.1	124	83.2	
Social support							
Poor	44	20.1	13	18.8	31	20.8	0.807
Average	132	60.7	43	62.3	89	59.7	
Good	42	19.2	13	18.8	29	19.5	
Education							
Undergraduate	96	44	7	10.1	89	59.7	0.017
Graduate	94	43.1	50	72.5	44	29.5	
Post-graduate	28	12.8	12	17.4	16	10.7	
Psychotropic medication							
No	192	88.1	60	87	132	88.6	0.812
Yes	26	11.9	9	13	17	11.4	
Psychiatric history							
No	210	96.3	65	94.2	145	97.3	0.362
Yes	8	3.6	4	5.8	4	2.7	

### Statistical analysis

The sample size was calculated for a 95% CI, with a tolerated margin error of 5% and an expected distress of 15%, using the formula N = Za^2^P(1-P)/d2 in which Za^2^ = 1.96, P = 0.15, and d2 = 0.0025. To set the goal, at least 100 surveys were needed to be completed [24]. The population was divided into two groups. One group included participants who completed the survey before November 2nd (before second lockdown) and the other group included participants who completed the survey after November 2nd (during the second lockdown). Statistical analysis consisted firstly in a descriptive and comparative analysis of participants’ sociodemographic characteristics using Chi-squared test for each categorical variable. Secondly, descriptive analyses were performed on severity categories for depression in children (MDI-C), and for depression, anxiety and stress in parents (DASS-21), and for perceived behaviors in children (CBCL). Differences between groups (before or during the second lockdown) regarding severity levels were tested using *X*^2^. Thirdly, Mann–Whitney U tests were performed to test differences for every outcome between groups. Binary logistic regression analyses were performed to estimate risk factors for moderate-to-severe depression in children. These were presented as odds ratios, with 95% confidence intervals, adjusting for confounders. All tests were two-tailed and alpha was set at 0.05. All analyses were performed using IBM SPSS^®^ v26.

## Results

### Sociodemographic characteristics

Information from 218 adults and 273 children was collected, as several children from one family responded to the questionnaires. Children were divided into 3 age categories: 82 children were aged 7–10 years old, 72 were 11–13 years old and 119 were 14–17 years old. Half were girls (50.6% girls, 49.4% boys). Parents were also divided into age categories: 46 parents were 0–39 years old, 78 were 40–45 years old, 69 were 46–51 years old and 25 were more than 51 years old. Most parents were European (90.8%). 121 parents reported no financial impact of the pandemic (55.7%) on their daily life. 56.6% lived in town and the others in the countryside. The majority of parents were in a relationship (82.6%). Regarding their education level, 44% of parents were undergraduate, 43.1% were graduate and 19.2% post-graduate. It should be noticed that parent education was different between before the 2nd lockdown versus during the 2nd lockdown, where more than half (59.7%) of parent-participants during the 2nd lockdown were undergraduate, Χ^2^ (2, N = 273) = 8.151, *p* = 0.017. 20.1% of parents reported having little social support. Finally, 88.1% of parents didn't take any psychotropic medication and 96.3% didn't have any psychiatric history. Except for parent education, no significant difference was found between groups.

### Severity of symptoms

Descriptive statistics showed that 17.9% of children presented moderate-to-severe depression scores (Table 2) This rate rose from 10.8% to 21% between before and during the second lockdown, χ^2^ (3, N = 274) = 12.07, *p* = 0.007. However, no difference between the two periods was found for each age category. Nevertheless, it should be noticed that differences based on age categories were found regardless the time period. Older adolescents reported higher scores reaching 5.9% of severe depressive symptoms for the 14–17 years old category, χ^2^ (3, N = 274) = 17.70, *p* = 0.007. Comparisons of the subgroup that completed the questionnaires before the second lockdown with the subgroup that completed them during lockdown showed a significant difference in the percent of children who endorsed moderate-to-severe depression symptoms. Only 53.2% of children did not have a depression score during the second lockdown.

**Table 2:** Severity Categories of Emotional Distress Measures for Children by Groups (*N* = 274)

Measurement	No (%)					
	Total	Time		Age		
		Before 2nd lockdown	During 2nd lockdown	7–10 years old	11–13 years old	14–17 years old
MDI-C						
Normal	163 (59,5)	62 (73,8)	101 (53,2)	64 (78,0)	39 (54,2)	60 (50,4)
Mild	62 (22,6)	13 (15,5)	49 (25,8)	12 (14,6)	19 (26,4)	32 (26,1)
Moderate	37 (13,5)	5 (6)	32 (16,8)	5 (6,1)	11 (15,3)	21 (17,6)
Severe	12 (4,4)	4 (4,8)	4 (4,2)	1 (1,2)	3 (4,2)	7 (5,9)

*MDI-C* Multiscore Depression Inventory for Children

As we observed in children, severity of parents’ scores worsened during the second lockdown. Almost half of the parents reported moderate-to-severe scores of depression during the second lockdown rising from 25% to 54.6%; χ^2^ (4, N = 218) = 36.86, *p* < 0.001 (Table 3). Four out of ten parents reported moderate-to-severe anxiety scores during the second lockdown rising from 19.2% to 40.7%, χ^2^ (4, N = 218) = 10.94, *p* = 0.027. Less than half of the parents reported moderate-to-severe stress scores during the second lockdown rising from 25% to 46.0%, χ^2^ (4, N = 218) = 22.67, *p* < 0.001.

**Table 3:** Severity Categories of Emotional Distress Measures for Parents by Groups (*N* = 218)

Measurement	Time			*p*
	Total	Before 2nd lockdown	During 2nd lockdown	
DASS-21				
Depression score				
Normal	82 (29,9)	45 (66,2)	37 (24,7)	0
Mild	37 (13,5)	6 (8,8)	31 (20,7)	
Moderate	61 (22,3)	13 (19,1)	48 (32)	
Severe	14 (5,1)	3 (4,4)	11 (7,3)	
Extremely severe	24 (8,8)	1 (1,5)	23 (15,3)	
Anxiety score				
Normal	129 (29,2)	51 (75)	78 (52)	0.027
Mild	15 (6,9)	4 (5,9)	11 (7,3)	
Moderate	33 (15,1)	5 (7,4)	28 (18,7)	
Severe	24 (11)	5 (7,4)	19 (12,7)	
Extremely severe	17 (7,8)	3 (4,4)	14 (9,3)	
Stress score				
Normal	100 (45,9)	46 (67,6)	54 (36)	0
Mild	32 (14,7)	5 (7,4)	27 (18)	
Moderate	44 (20,2)	6 (8,8)	38 (25,3)	
Severe	31 (14,2)	10 (14,7)	21 (14)	
Extremely severe	11 (5)	1 (1,5)	10 (6,7)	

*DASS-21* 21-item Depression Anxiety and Stress Scale

Considering the CBCL scale, more than a third of the youth (35.3%) reached a subclinical-to-clinical threshold of internalized symptoms (anxio-depression, withdrawal or somatic symptoms) and 12% of externalized problems (aggressive or delinquent behaviors). Chi-squared tests showed that during the second lockdown, clinical thresholds for internalized disorders are more often reached than before the second lockdown (22.7% before, 42.6% after), χ^2^ (4, N = 243) = 9.91, *p* = 0.007. This increase is not significant for externalized disorders; parents reported more externalized symptoms but without reaching clinical thresholds.

Mann-Withney test showed a statistical difference for DASS-21 and MDI-C results when comparing before and during the second lockdown, reporting an increase in depression and anxiety scores for children and their parents (Table 4).

**Table 4:** Medians and Interquartile Ranges for Emotional Distress Scores in Children and Parents by Time

Measurement	Median (IQR)			
	Total	Time		*p*
		Before 2nd lockdown	During 2nd lockdown	
MDI-C (*N* = 274)				
Depression	16.0 (8.8–28.0)	12.0 (5.0–18.8)	18.0 (10.8–31.0)	< .001
DASS-21 (*N* = 218)				
Depression	12.0 (6.0–18.0)	6.0 (2.0–13.5)	14.0 (9.5–20.0)	< .001
Anxiety	4.0 (2.0–12.0)	2.0 (0.0–7.5)	6.0 (2.0–14.0)	< .001
Stress	16.0 (10.0–22.0)	12.0 (8.0–19.5)	18.0 (12.0–24.0)	< .001
CBCL (*N* = 243)				
Internal	61.0 (52.0–67.0)	57.5 (48.0–63.0)	63.0 (54.0–69.0)	< .001
External	53.0 (46.0–59.0)	51.0 (44.0–58.0)	54.0 (48.0–59.0)	.039

*MDI-C* 79-item Multiscore Depression Inventory for Chi ldren, *DASS-21* 21-item Depression, Anxiety and Stress Scale, *CBCL* Chi ld Behavior Checkl ist

### Correlations

Pearson’s correlations were performed, showing that children’s MDI-C scores are significantly positively correlated for each score of DASS-21 scale (Pearson’s correlation are 0.401, 0.436 and 0.331 between MDI-C scores and anxiety, depression and stress DASS-21 scores, respectively).

### Risk factors

We also studied which factors, including parents’ age, children’s age and gender, social support, civil situation, living environment, psychotropic medication, psychiatric parental history, and depression, anxiety and stress scores could be risk factors for higher depression scores in children (Table 5). These risk factors are presented as odds ratios, adjusting for parents’ education level, financial impact, and ethnic origin as confounders. Logistic regression analysis showed that psychotropic medication use by parents (OR, 4.110; 95% CI, 1.252–13.486; *p* = 0.020) and parents’ depression scores (OR, 1.087; 95% CI, 1.002–1.180; *p* = 0.045) were associated with moderate-to-severe levels of depression in children. In other words, if parents take psychotropic medication, children are 4.22 times more likely to have a higher level of depression and are 9% more likely to have a higher level of depression if parents are also depressed.

**Table 5:** Adjusted Risk Factor for Child Depression

Measurement	N	OR (95% CI)	Category *p*-value	Overall *p*-value
Children’s age				
7–10 years old	66	0,287 (0,075–1,108)	0.07	0.067
11–13 years old	54	1,489 (0,545–4,068)	0.437	
14–17 years old	98	Reference	NA	
Children's gender				
Girls	108	0,654 (0,266–1,608)	0.355	0.355
Boys	110	Reference	NA	
Parents’ age				
0–39 years old	46	0,212 (0,40–1,110)	0.066	0.146
40–45 years old	78	0,252 (0,068–0,938)	0.04	
46–51 years old	69	0,261 (0,073–0,926)	0.038	
51 +	25	Reference	NA	
Living environment				
Countryside	94	1,174 (0,487–2,831)	0.721	0.721
Town	124	Reference	NA	
Civil situation				
In a relationship	180	1,007 (0,348–2,915)	0.99	0.99
Single	38	Reference	NA	
Social support				
Poor	44	2,349 (0,572–9,649)	0.236	0.334
Average	132	1,131 (0,335–3,819)	0.843	
Good	42	Reference	NA	
Psychotropic medication				
Yes	26	4,110 (1,252–13,486)	0.02	0.02
No	192	Reference	NA	
Psychiatric history				
Yes	8	2,117 (0,284–15,798)	0.465	0.465
No	210	Reference	NA	
DASS-21 score				
Depression score		1,087 (1,002–1,180)	0.045	
Anxiety score		0,994 (0,918–1,075)	0.876	
Stress score		1,032 (0,952–1,119)	0.447	

Adjusted for financial impact, ethnic origin and parental education

*DASS-21* 21-item Depression, Anxiety and Stress Scale

## Discussion

This cross-sectional study aimed to assess the prevalence of mental health issues among parents and their children and to compare children’s and parents’ mental health before and during the second national lockdown in Belgium during the COVID-19 pandemic. One of the strength of our study is that these outcomes were assessed with validated instruments for adult and pediatric populations. The first key finding was the high rate of depressive symptoms in children. The youth self-questionnaire MDI-C showed that more than 4% of our sample presented clinical scores of major depression. This result is higher than the prevalence usually found in the general population in other western European countries: in 2009, Merikangas reported a prevalence of major depressive disorder of 0.6% for 5 to 16 years old children in Great Britain [25]. The rate of moderate-to-severe depressive symptoms rose to 17.9% in our sample. Specifically, we found that older children, and especially adolescents (14–17 years old), had higher scores of depression. Half of them reported mild-to-severe levels of depression compared to 22% of younger children (7–10 years old). This could be related to the specific developmental stage of adolescence: the teenage acquires his autonomy and becomes individualized from his parents while younger children are more dependent on their parents. During lockdown, the adolescent loses his autonomy and social contacts at a time when he particularly relies on socialization with peers. He must now avoid conflict with his parents with whom he is confined. At the age of becoming an adult, he does not know what his future will be like. Of course, puberty and hormonal changes are also part of the reason why adolescents are more vulnerable than younger children [26, 27].

We also observed a significant increase in depression scores between the two periods to the point that less than half of the children still showed no signs of depression during the second lockdown. The proportion of children with severe depressive symptoms remained stable but the rate of children with mild-to-moderate depressive symptoms strongly increased, at the expense of the proportion of children who did not show depressive symptoms. These data draw our attention to the impact of the pandemic on all of society and not just vulnerable patients. Rothe et al. hypothesized that children with a mental health condition could previously be disconnected from school and society and had then less suffered from social restrictions or had developed strategies to cope with strained relationships (for example consulting a specialist) [28]. Other authors found that the mental health of some in more marginalised groups improved with the closure of schools, suggesting some protection against harmful expectations and relationships at school [29].

Parents’ perception of their children’s mental health, according to the Child Behavior Checklist, showed an increase in internalized disorders (anxio-depression, withdrawal or somatic symptoms) during the second lockdown, supporting the interest of being attentive to more silent symptoms. Thus, parents seemed to capture changes in their children’s mental health as the children self-reported it. The fact that internalized symptoms are more often detected by parents than externalized disorders could be related to the home confinement itself, the need to live together in a certain understanding and the proximity between family members. Bera et al. hypothesized that externalized disorders were more likely to occur in adolescents with pre-existing behavioural disorders and emerged mostly during later waves of the pandemic [30].

Among parents, depression scores are soaring: almost half of the adult population reported moderate-to-severe symptoms of depression. As for their children, parents’ depression scores more than doubled between the two evaluation periods. Interestingly, we can observe a correlation between parental scores of depression, anxiety and stress with the depression scores of their children at a significant level. It confirms previous studies showing that the higher the parents’ individual stress is, the more psychological problems the children have [31]. This can be explained by the lack of psychological availability from parents who were already preoccupied by their own health and financial situation during the crisis and showed less support and warmth towards their children [32]. Because of higher parental distress, conflicts between parents and their children may easily escalate, worsening children's feelings and their well-being. These results can be understood in both directions: parents can also be distressed and depressed by the psychological difficulties of their children. Fosco et al. emphasises the importance of promoting family interventions to help them maintain cohesion and manage conflicts to support child adjustment. [33] Indeed, decreases in family cohesion and increases in family conflict predicted subsequent child maladjustment and the development of internalized and externalized disorders.

In terms of risk factors, our results highlighted that parents who use psychotropic medication and report higher depression scores were associated with worse depression scores in children. Parents’ mental health thus appears to be an independent risk factor for worse mental health outcomes in children. Parents could be overwhelmed by their own mental problems and be less careful to those of their children. Some authors showed that people living with a mental health illness were concerned about disruption of services and running out of medication [34], in addition to fears of being more severely affected by COVID-19 due to co-morbidities associated with the side effects of psychotropic drugs (obesity, diabetes). Henceforth, taking medication suggests that parents previously had mental health issues which could be worsened by restrictions. They may have experienced difficulties in obtaining their medication (especially adults under injectable drugs) or considered that psychotropic medication was a risk for developing more severe COVID-19 infection. In addition, as we know, environmental stress events can throw a stabilised disease off balance (loss of a daily rhythm, increased tobacco/alcohol consumption, economical stress, interruption of outpatient care).

## Summary

In Belgium, we experienced two strict lockdowns: from March to May 2020 and from November 2020 to February 2021. When comparing depression and anxiety scores before and during the second lockdown, we noticed that the second lockdown worsened the effects on children’s and parents’ mental health. Scores in MDI-C were suggestive of depression in almost half of children while more than half of parents presented symptoms of depression. We found an increase in the proportion of children with mild-to-moderate depressive symptoms at the expense of the proportion of children without any depressive disorder, while the category of children with severe symptoms remained stable. Therefore, these observations do not argue for a worsening of the emotional state of already depressed children but rather a general deterioration. Adolescents suffered more from the lockdown than children.

Our study highlighted the correlation between children's and parents' depression. Given that parents with depressive symptoms have doubled since the beginning of the second lockdown, it seems crucial that mental health promotion targets both children and their parents to achieve better outcomes. A particularly fragile population is likely to be parents taking psychotropic medication. It seems then that adult and child psychiatry are linked and must be considered together. Because we couldn’t follow the participants throughout the year to conduct a longitudinal study, these results have to be considered carefully.

Health measures taken to control COVID-19 pandemic must include concern about the youth’s mental health and have to be thought out in the mid-term to avoid iterative lockdowns that worsen depressive symptoms for both children and parents. Further studies are needed to assess consequences of the pandemic and the restrictive measures in the long-term from a developmental perspective.

## Limitations

This study used a cross-sectional comparative design between two independent samples, making test–retest impossible. We focused on a non-clinical population and compared epidemiological data between two samples of different people, which limits causality effect. Moreover data were collected over an extended period to get a large sample; this temporality involves potentially changing variable. Limitations also include the electronic way of filling in the questionnaire, preventing children from being accompanied throughout the questionnaires and possible diffusion bias. Indeed, even if the questionnaires were distributed by official communication channels, the circulation of the survey included familiar and professionals contacts. Although the length of the scales can bring a more accurate assessment of the child’s condition, motivation is needed to reach the end of the questionnaire and can be missing in very depressed children, recall the low response rate. At last, given our medium sample size and differences in parents’ education between groups, the generalizability of results has to be considered with caution but give a good indication of how children are coping in times of confinement. Further longitudinal studies on larger samples are needed to generalize these results and to evaluate the cumulative effect of iterative lockdowns on children’s mental health.

## Data Availability

The datasets generated and analysed during the current study are stored in the REDCap© database located on the leading site, Queen Fabiola Children’s University Hospital (HUDERF) and available on request.

## Abbreviations

Covid-19: Coronavirus disease
DASS-21: Depression, anxiety and stress scale
MDI-C: Multiscore depression inventory for children
CBCL: Child behavior check list
